# Development of Antimicrobial Nitric Oxide-Releasing Fibers

**DOI:** 10.3390/pharmaceutics13091445

**Published:** 2021-09-10

**Authors:** Daniel C. Wang, Justin R. Clark, Richard Lee, Adam H. Nelson, Anthony W. Maresso, Ghanashyam Acharya, Crystal S. Shin

**Affiliations:** 1 Department of Surgery, Baylor College of Medicine, Houston, TX 77030, USA; dw16@bcm.edu (D.C.W.); richard.lee@bcm.edu (R.L.); adam.nelson@bcm.edu (A.H.N.); 2 Department of Molecular Virology and Microbiology, Baylor College of Medicine, Houston, TX 77030, USA; jrclark@bcm.edu (J.R.C.); maresso@bcm.edu (A.W.M.)

**Keywords:** nitric oxide, electrospun fibers, biopolymers, antimicrobial, NONOate

## Abstract

Nitric oxide (NO) is a highly reactive gas molecule, exhibiting antimicrobial properties. Because of its reactive nature, it is challenging to store and deliver NO efficiently as a therapeutic agent. The objective of this study was to develop NO-releasing polymeric fibers (NO-fibers), as an effective delivery platform for NO. NO-fibers were fabricated with biopolymer solutions of polyvinyl pyrrolidone (PVP) and ethylcellulose (EC), and derivatives of *N*-diazeniumdiolate (NONOate) as NO donor molecules, using an electrospinning system. We evaluated in vitro NO release kinetics, along with antimicrobial effects and cytotoxicity in microorganisms and human cell culture models. We also studied the long-term stability of NONOates in NO-fibers over 12 months. We demonstrated that the NO-fibers could release NO over 24 h, and showed inhibition of the growth of Pseudomonas aeruginosa (P. aeruginosa) and methicillin-resistant Staphylococcus aureus (MRSA), without causing cytotoxicity in human cells. NO-fibers were able to store NONOates for over 12 months at room temperature. This study presents the development of NO-fibers, and the feasibility of NO-fibers to efficiently store and deliver NO, which can be further developed as a bandage.

## 1. Introduction

The delivery of gases has attracted great attention from researchers over the years, with its potential applications ranging from medicine to the environment and energy storage [1]. The use of highly porous solid materials (e.g., zeolites, metal–organic frameworks, polymers) for storage and subsequent delivery has been explored recently, as they offer many advantages compared to storing gases in a bottle or tank [2,3]. Specifically, these porous materials allow for an increase in storage capacity, owing to their high surface-to-volume ratio, as more gas can be stored within a given volume of solid than in a tank under high pressure [1]. In addition, it was found that a small volume of gases is easier to handle when stored in solid materials. Moreover, the use of solid materials allows for the delivery of gases, which avoids the systemic side effects that are often associated with delivery from a tank [1,4,5]. For these reasons, highly porous solid materials, particularly polymers, are considered to be an effective delivery platform for nitric oxide (NO).

NO is a free radical gas molecule that is endogenously produced in mammals by the enzyme nitric oxide synthase. NO plays a key role in the innate immune response against foreign organisms, including bacteria [3,6,7]. The reaction of NO with oxygen spontaneously produces reactive nitrogen oxide species (RNOS), which subsequently cause oxidative and nitrosative damage to bacteria cells, through alteration of DNA, inhibition of enzyme function, and induction of lipid peroxidation. Due to the multiple, simultaneous antimicrobial mechanisms by NO, most bacteria are less likely to develop significant resistance or tolerance, which makes NO an ideal antimicrobial agent [8]. However, the current use of NO is hindered by its high chemical reactivity and limited water solubility, making it difficult to use in biomedical applications.

The development of small-molecule NO donors, such as *N*-diazeniumdiolates (NONOate), offers a promising solution to this problem, as these donor molecules are able to stabilize chemically, and subsequently release NO when required [9]. Each NONOate molecule contains a diolate group that can spontaneously release two NO molecules upon contact with moisture. Because of the spontaneous action of NO, most bacteria are unable to develop clearance mechanisms against NONOates [10]. Additionally, NONOates can be chemically modified with functional groups, to increase the half-lives of NONOates for up to 20 h, further improving the stability of NO [11]. Although the premature release of NO has been a concern, the use of polymers as a storage and delivery system has been shown to stabilize NO, thereby allowing for the controlled delivery of NO [12].

For this reason, the use of polymers is believed to be an effective method in harnessing the antimicrobial properties of NO, since the treatment of topical infections involves the local delivery of NO to injured areas [13]. Therefore, we hypothesized that the development of a polymer-based delivery system would improve the efficiency of NO delivery and maximize the potential of NO as an antimicrobial agent, thus broadening its possible applications, such as a bandage. In this study, we developed NO-releasing polymeric nanofibers (NO-fibers), using biocompatible polymers and NONOates in a simple electrospinning process. Thus, the developed NO delivery system can (1) protect NO from moisture, which releases NO in a sustained manner; and (2) inhibit microbial growth without causing cytotoxicity in human cells.

## 2. Materials and Methods

### 2.1. Materials

All chemicals used in this study, including polyvinylpyrrolidone (PVP; MW 360 kDa) and ethylcellulose (EC; 48% ethoxyl basis), were purchased from Sigma-Aldrich (St. Louis, MO, USA) unless noted otherwise. Proline (PROLI)–NONOates, dipropylenetriamine (DPTA)–NONOates, and diethylenetriamine (DETA)–NONOates, and nitrate/nitrite colorimetric assay kits were purchased from Cayman Chemical Company (Ann Arbor, MI, USA). *Pseudomonas aeruginosa* (PAO1 strain) and methicillin-resistant *Staphylococcus aureus* (MRSA; USA300 strain) were generously provided by Dr. Anthony Maresso at Baylor College of Medicine (Houston, TX, USA). Human microvascular endothelial cells and human dermal fibroblasts, cell culture media and supplements, mini dialysis tubes, and alamarBlue cell viability assay kits were purchased from Thermo Fisher Scientific (Waltham, MA, USA).

### 2.2. Fabrication of Electrospun NO-Fibers

For the electrospinning process, we first prepared polymer solutions containing NONOates. Briefly, 10% PVP and 8.5% EC solutions were prepared by dissolving PVP or EC in ethyl alcohol with mechanical stirring and heating at 60 °C. Ten milligrams of NONOates, PROLI–, DPTA– and DETA–NONOates were individually dissolved in sodium hydroxide (10 mM, NaOH) solution and then added to 20 mL of PVP or EC solution to yield 2.3–3 mM NONOate concentrations. The resulting solutions were vortexed sufficiently and then transferred into a cartridge to fabricate NO-fibers.

A desktop electrospinning system (4SPIN, Contipro, Dolní Dobrouč, Czech Republic) is a unique system where the collector is placed above the emitter. This setup prevents accidental spoilage of nanofiber sheets because of the dripping polymer solutions on the collected nanofibers during the spinning process. The tip of the syringe containing the biopolymer solution was connected to a single jet emitter, where the high electric voltage was applied. The polymer solution containing NONOates was injected at a rate of 30 µL per minute and emitted through the injector tip (19 gauge) upon applying a high voltage (10–15 kV). The emitted fibers were deposited on a grounded rotating collector at 1000 rpm. The distance between the emitter and the collector was maintained at 10 cm. The collected nanofibers containing NONOates, NO-fibers, were carefully separated from the collector and then cut into circular samples 8 mm in diameter for in vitro evaluations. Six-to-eight NO-fibers of each type were kept in air-tight containers to prevent moisture exposure until further evaluations.

For scanning electron microscope (SEM) imaging, we used a field emission SEM (SU8230, Hitachi, Japan). NO-fibers were cut into small pieces, 2 × 2 mm, then placed on carbon tape mounted on an aluminum stub. The samples were sputter-coated with gold/palladium at 4 nm thickness (Leica EM ACE600, Leica Microsystems, Wetzlar, Germany). Images were obtained using a secondary electron detector, 5–20 kV, and at a 9 mm working distance. Diameters of NO-fibers were measured from obtained images using ImageJ software (National Institute of Health, Bethesda, MD, USA). At least 12 fibers were measured from three images.

### 2.3. In Vitro Evaluation of NO Release

A release study was performed in triplicate on each fabricated NO-fiber in order to evaluate NO release kinetics. To trigger the NO release from NO-fibers, each NO-fiber sample was placed in a mini dialysis tube (MWCO: 2000Da) with 500 µL of phosphate-buffered saline (PBS). The mini dialysis tube was then placed in a 5 mL centrifuge tube also containing 500 µL of PBS. Samples were maintained at 37 °C in an orbital shaker (200 rpm; Fisher Scientific). PBS was collected and replaced with an equivalent amount of fresh medium at the following times: 5 min, 10 min, 30 min, 60 min, 90 min, 2 h, 24 h. Collected samples were evaluated using a nitrite colorimetric assay. Absorbance was measured at 540 nm via a microplate reader (CLARIOstar, BMG Labtech, Ortenberg, Germany). Absorbance values of standard wells were plotted to obtain a nitrite standard curve.

We determined the total NO content from the NO-fibers. Each NO-fiber was dissolved in 250 µL of EtOH, followed by 250 µL of PBS. For EC NO-fibers, solutions were centrifuged at 13,000× *g* for 10 min to collect the supernatant. Samples were then analyzed using a nitrite colorimetric assay to determine the total NO content. The NO release was determined as follows:

(1) $$\mathrm{NO}\;\mathrm{released}\;(\%)=\frac{\mathrm{NO}\;\mathrm{released}}{\mathrm{Total}\;\mathrm{NO}\;\mathrm{content}}\;\times \;100$$

### 2.4. Evaluation of Antimicrobial Effect of NO-Fibers

*Pseudomonas aeruginosa* and methicillin-resistant *Staphylococcus aureus* (MRSA) stocks were kept frozen at −80 °C until required for experiments. Two days before the experiment, each strain was streaked on a Luria broth (LB) agar plate and incubated at 37 °C overnight. A single colony was picked and inoculated in LB broth. MRSA and *P. aeruginosa* were cultured as planktonic cells in LB broth at 37 °C overnight. Bacteria were then diluted in LB broth (1:100) until the optical density (OD) at 600 nm reached 0.3–0.6 (i.e., the logarithmic phase). The bacterial culture was then appropriately diluted to 1 × 10^7^ CFU/mL in LB broth then the culture was transferred to each well in a 96-well plate. To each well, one NO-fiber sample was added. The plate was incubated at 37 °C, and the OD at 600 nm was measured for 24 h at 15-min intervals (CLARIOstar, BMG LABTECH GmbH, Ortenberg, Germany). Fresh LB and LB with one NO-fiber sample were added to wells as controls to determine possible interference; the presence of NO-fibers did not affect OD as NO-fibers became transparent upon hydration. The growth curve was plotted over 24 h, and the final OD values at 24 h were used to determine the viability. The percent viability was determined as follows:

(2) $$\mathrm{Viability},\;\%=\frac{\mathrm{OD},\;\mathrm{treated}\;\mathrm{with}\;\mathrm{NO}\_\mathrm{fibers}}{\mathrm{OD},\;\mathrm{untreated}\;\mathrm{control}}\;\times \;100$$

### 2.5. In Vitro Evaluation of Cytotoxicity

The cytotoxicity of NO-fibers was evaluated in human dermal fibroblast cells (HDFs) and human microvascular endothelial cells (HMVECs). HDFs and HMVECs were maintained at 37 °C with 5% CO_2_ in medium 106 supplemented with low-serum growth supplement and medium 131 supplemented with microvascular growth supplement, respectively. Cells were cultured as monolayers in tissue-culture treated 75 cm^2^ flasks. Once confluency levels reached 85%, cells were trypsinized and passaged. HDFs used in the evaluation were between passages 3 and 6. HMVECs used in the evaluation were between passages 5 and 7.

HDFs/HMVECs were seeded in each well of a 24-well plate (10^4^ cells per well). Once cells were added, the plate was incubated overnight in order for cells to adhere to the surface of the wells. One NO-fiber sample was added to each well using a Transwell insert, and the plate was incubated for 24 h. Transwell inserts were used to ensure complete removal of NO-fibers before performing viability assay to prevent interference with absorbance values. After 24 h, NO-fibers were removed from wells, and cell viability was evaluated via alamarBlue cell viability assay. Absorbance was measured at 570 nm via a microplate reader (CLARIOstar; BMG Labtech). Wells without films were used as control.

### 2.6. Evaluation of Stability of NO-Fibers

NO-fibers were stored at 25 °C in sealed, air-tight, containers for 12 months to evaluate the long-term stability. We used the total NO content obtained from the NO release kinetic studies as initial total NO content to determine the stability. Each NO-fiber was dissolved in 250 µL of EtOH, followed by 250 µL of PBS. Samples were then analyzed using a nitrite colorimetric assay to determine the total NO content after 12 months. The remaining NO content was calculated as follows:

(3) $$\mathrm{Remaining}\;\mathrm{NO}\;\mathrm{content}\;(\%)=\frac{\mathrm{Total}\;\mathrm{NO}\;\mathrm{content}\;\mathrm{at}\;t=12\;\mathrm{months}}{\mathrm{Initial}\;\mathrm{total}\;\mathrm{NO}\;\mathrm{content}\;}\;\times \;100$$

### 2.7. Statistical Analysis

All experiments were performed with at least three samples. Data are shown as mean ± standard deviation. Statistical analyses were performed by an unpaired, two-tailed Student *t*-test using GraphPad Prism 8.0 software (GraphPad Incorporation, San Diego, CA, USA). Moreover, *p* values < 0.05 were considered to be statistically significant.

## 3. Results and Discussion

### 3.1. Fabrication of Electrospun NO-Fibers

We used electrospinning technology to fabricate NO-releasing polymer-based nanofibers. Electrospinning is a highly reproducible process that produces polymeric fibers ranging from a few nanometers to micrometers, and with a high surface-to-volume ratio. Furthermore, it allows for easy control of the mechanical properties of the electrospun fibers, such as porosity, which can be easily controlled by manipulating various processing parameters. Specifically, the physical properties of the fibers can be adjusted by altering the polymer concentration, the voltage, and the speed of solution injection.

For the successful delivery of NO, the selection of an appropriate biopolymer and NO donor molecule was crucial. The ideal candidate for the biopolymer needs to be highly biocompatible, yield a soft, yet easy-to-handle, final product, and prevent premature NO release. To evaluate the suitable biopolymers as a NO delivery system, we selected PVP and EC as biopolymers to fabricate NO-fibers. PVP and EC are well-characterized synthetic biopolymers that are widely used in biomedical and pharmaceutical research, as well as nanofiber fabrications. PVP has been used as a plasma volume expander and can also be found in personal hygiene products and pharmaceutical applications [14]. EC is a cellulose-derived synthetic biopolymer found in pharmaceutical formulations as an excipient [15]. Both PVP and EC are non-ionic and physiologically inert, but PVP is hydrophilic, while EC is hydrophobic. Despite the difference in polymer–water interactions, PVP and EC are readily dissolved in ethyl alcohol, which was the rational choice of solvent to protect NONOates from water. It was necessary to avoid using water as a solvent during the NO fabrication processes. However, it is worth noting that NONOates are stable in alkaline solutions such as NaOH.

NONOates, along with S-nitrosothiols, are commonly used NO donor molecules, to overcome the limitation of gaseous NO delivery. For S-nitrosothiol molecules, NO is bound to a thiol group and is released upon bond cleavage in the presence of a trigger, specifically metal ions, ascorbate, or light. Thus, while S-nitrosothiol can release NO in a physiological environment, it can only do so under specific conditions [16,17,18]. Moreover, S-nitrosothiols can only release one molar equivalent of NO per S-nitrosothiol molecule, and many target pathogens have precise mechanisms that degrade the donor molecule before NO release is possible [19]. For this reason, NONOates were selected over S-nitrosothiols as the NO donor molecule for NO-fibers. NONOates are able to release two equivalents of NO per molecule, and most bacteria have not developed clearance mechanisms against NONOates, making NONOates a better candidate as an antimicrobial agent [10].

The current polymeric delivery vehicles for NONOates commonly involve microparticles or nanoparticles, due to their customizability, which allows for distinct release kinetics and targeted delivery [12]. The preparation of such platforms often requires the treatment of biocompatible polymers with NO gas under high pressure, in order to functionalize the polymeric backbone with NONOates [20]. Though such a method of preparation has been successful, covalent coupling of NONOates can be rather complex and requires the use of high-pressure NO tanks, which can be rather costly. The electrospinning process, on the other hand, is a straightforward and economical method that can also be used to fabricate polymeric fibers for drug delivery applications [21,22,23].

**Figure 1:**
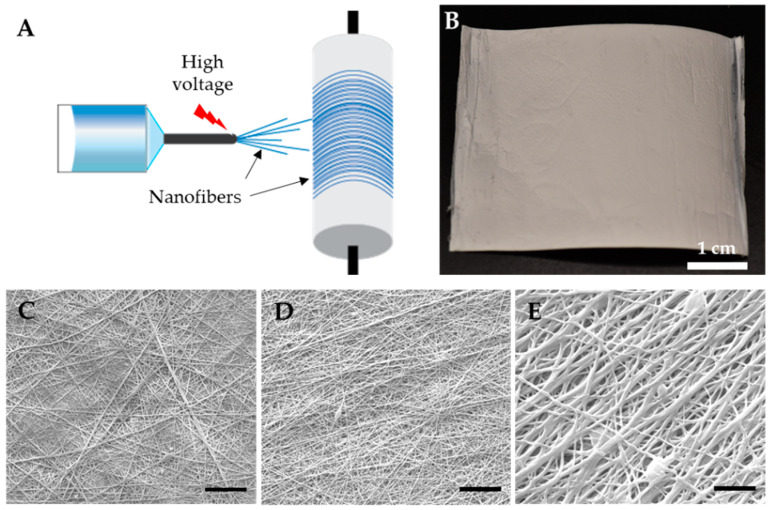
Fabrication of NO-fibers. (**A**) Schematic of electrospinning of NO-fibers. The polymer solution is emitted through the needle when high voltage is applied to the tip of the needle and fibers are collected on a rotating collector; (**B**) photograph of an electrospun sheet of PVP NO-fibers. Scanning electron microscope images of PVP NO-fibers: (**C**) PROLI–NO-fibers; (**D**) DPTA–NO-fibers; (**E**) DETA–NO-fibers. Scale bar: 5 µm.

Polymeric fibers were formed, as the solvent evaporated when the high voltage was applied (Figure 1A). The emitted fibers were deposited on a rotating collector over 12 h, as a white sheet. A rotating collector was used, since it allows for better alignment of the nanofibers compared to the nanofibers that are deposited on a static collector. The NONOates containing NO-fibers were then carefully separated from the collector, and then cut into smaller pieces (Figure 1B). To examine the nanofibrous structure of NO-fibers, we used a scanning electron microscope to visualize them. The SEM images revealed that nanofibers successfully formed from electrospinning PVP solutions containing PROLI–, DPTA–, and DETA–NONOates (Figure 1C–E). It showed that the average diameters of the PROLI–, DPTA–, and DETA–NO-fibers were 278 ± 93 nm, 222 ± 83 nm, and 358 ± 143 nm, respectively.

### 3.2. In Vitro Release of NO-Fibers

#### 3.2.1. Effect of Biopolymers on Release Kinetics

**Figure 2:**
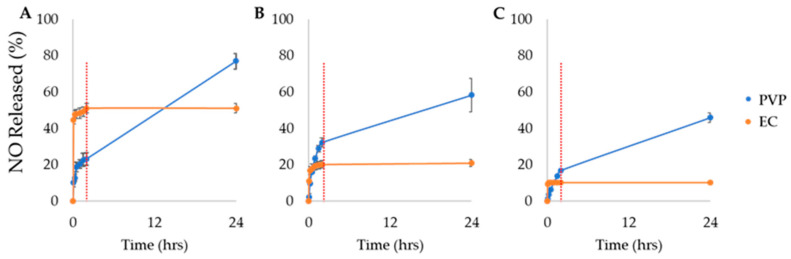
In vitro release of NO from NO-fibers fabricated with PVP or EC over 24 h. The percent NO released from NO-fibers containing PROLI–NONOates (**A**), DPTA–NONOates (**B**), and DETA–NONOates (**C**). Red lines indicate 2 h marks. Data are shown as mean ± standard deviation (SD).

In our study, we investigated the effect of biopolymers and NO donors on NO release, by fabricating NO-fibers using two different biopolymers and three types of NONOates. We evaluated the in vitro NO release from NO-fibers in PBS at 37 °C to simulate the physiological temperature. Additionally, the NO release is temperature-dependent [10,11]. The determination of NO content released from NO-fibers was performed using a colorimetric assay kit. As reported in Figure 2, we presented the NO release with respect to the total NO content in NO-fibers, as we calculated the amount of NO released using Equation (1). We observed that NO-fibers fabricated with PVP or EC were able to store and release NO over time. However, the aqueous solubility of biopolymers affected the overall release kinetics, since it was shown that NO-fibers fabricated with PVP released a higher content of NO over 24 h than EC NO-fibers (Figure 2). PVP NO-fibers immediately began to hydrate upon contact with PBS, releasing NO, and continued to release NO. PVP NO-fibers slowly dissolved in PBS, and the dissolution of the fibers freed NONOates to the aqueous environment, ultimately releasing NO for up to 24 h.

On the other hand, NO-fibers fabricated with EC exhibited immediate NO release within the first 30 min, and no detectable or minuscule amount of NO was released after 2 h. This was attributed to the insolubility of EC in aqueous solvents; NONOates present on the surface of EC NO-fibers instantly released NO when exposed to PBS. For NONOates encapsulated in EC fibers, the water molecules were not able to reach the NONOates and trigger NO release, since the EC NO-fibers did not disintegrate in PBS, due to EC’s hydrophobic nature.

We then determined the total NO content from each NO-fiber using the colorimetric assay kit. To understand the effect of biopolymers on release kinetics, we prepared NO-fibers with the same amount of NONOates. We first dissolved both PVP and EC NO-fibers in EtOH, then added PBS to release NO from NONOates. Then, we immediately measured the total NO content by the colorimetric assay. The total NO contents obtained for PROLI NO-fibers were 1.6 ± 0.07 µmol/mL for PVP and 2.8 ± 0.04 µmol/mL for EC. For DPTA NO-fibers, the total NO contents were 4.6 ± 0.3 µmol/mL and 6.7 ± 0.2 µmol/mL for PVP and EC, respectively. The total NO contents were 4.8 ± 0.25 µmol/mL for DETA-PVP NO-fibers and 8.1 ± 0.9 µmol/mL for DETA-EC NO-fibers. The NO encapsulation efficiency was higher in EC NO-fibers than PVP NO-fibers for each NONOate (Table 1). Interestingly, the measured total NO contents were higher in EC NO-fibers than PVP NO-fibers, even though the same amount of NONOates were added to each solution. The differences may suggest that NONOate molecules were not fully released from PVP. Even though PVP NO-fibers visibly disintegrated in both EtOH and water, it is possible that a high molecular weight of PVP might have required a longer dissolution time to release NO completely.

#### 3.2.2. Effect of NONOates on Release Kinetics

In addition to the effect of biopolymers, we evaluated the effect of various NONOates on the NO release kinetics. For our study, we evaluated the effect of different NONOates on in vitro release (Figure 2). Three NONOates with different functional groups, PROLI, DPTA, and DETA, were selected and incorporated into NO-fibers. These chemically modified NONOates exhibit half-lives of 2 s, 3 h, and 20 h, for PROLI–, DPTA–, and DETA–NONOates, respectively, at 37 °C and pH 7.4. The half-lives of NONOates are temperature-dependent, as the half-lives decrease under physiologically relevant conditions (37 °C), compared to the half-lives at room temperature (22–25 °C) [24,25,26]. The evaluation of release kinetics showed that approximately 50% of the total NO content was released from PROLI-EC NO-fibers within the first 24 h, while DPTA and DETA-EC NO-fibers only released approximately 20% and 10%, respectively. For PVP NO-fibers, within the first 24 h, PROLI-PVP NO-fibers released approximately 80% of its total NO content, while DPTA-PVP NO-fibers released only 60%, and DETA-PVP released even less, with 50%. (Figure 2). Because of the differences in half-lives, we expected that the release kinetics of NO would vary. As observed in Figure 2A, the percent of NO released within the first 24 h was the greatest for PROLI NO-fibers. This was due to the extremely short half-life of PROLI–NONOates. We also observed that the NO release was less from DETA NO-fibers than DPTA NO-fibers, most likely because the half-life of DETA–NONOates is much longer than DPTA–NONOates.

### 3.3. In Vitro Antimicrobial Activity of NO-Fibers

**Figure 3:**
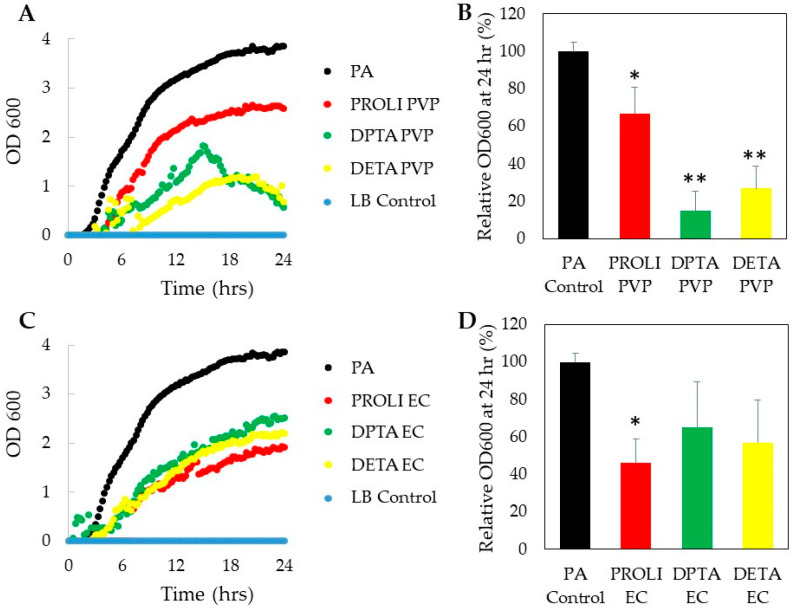
Antimicrobial effect of NO-fibers on *P. aeruginosa*, listed as PA. Growth curves of *P. aeruginosa* treated with PVP NO-fibers (**A**) and EC NO-fibers (**C**) over 24 h measured as optical density at 600 nm. The viability of *P. aeruginosa* at 24 h was compared to the untreated control following treatment with PVP NO-fibers (**B**) and EC NO-fibers (**D**). Data are shown as mean ± standard deviation (SD). *n* = 3 statistical significance: * *p* < 0.05, ** *p* < 0.01.

**Figure 4:**
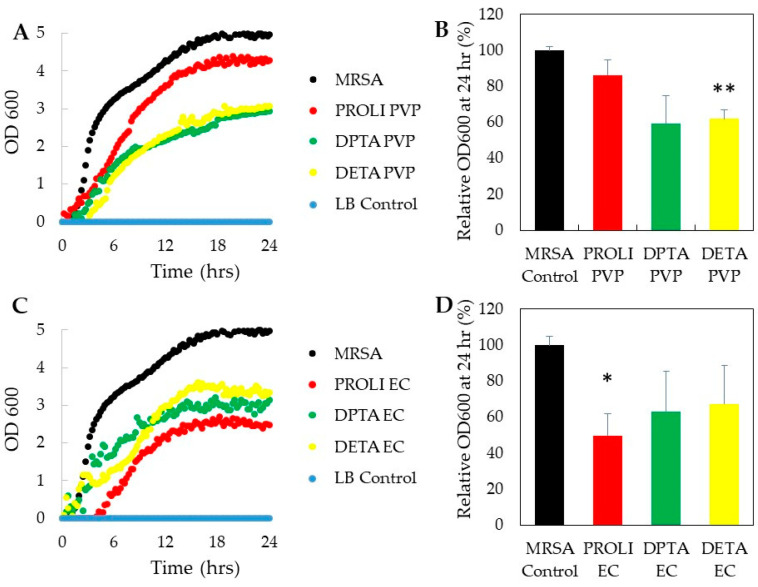
Antimicrobial effect of NO-fibers on MRSA. Growth curves of MRSA treated with PVP NO-fibers (**A**) and EC NO-fibers (**C**) over 24 h were measured as optical density at 600 nm. The viability of MRSA at 24 h was compared to the untreated control following treatment with PVP NO-fibers (**B**) and EC NO-fibers (**D**). Data are shown as mean ± SD. *n* = 3 statistical significance: * *p* < 0.05, ** *p* < 0.01.

Once we determined the NO release kinetics of each NO-fiber, we then studied the antimicrobial activity of NO-fibers in vitro. After treating with NO-fibers, we evaluated the toxicity on planktonic cultures of two microorganisms, *Pseudomonas aeruginosa* and methicillin-resistant *Staphylococcus aureus* (MRSA). Planktonic bacteria were used for this study, since they are commonly used to evaluate the antimicrobial efficacy against soft- tissue wound infections [27]. We measured the optical density (OD) at 600 nm to obtain the growth curves over 24 h, and the viability of bacteria treated with NO-fibers was compared to the untreated control group, according to Equation (2) (Figure 3 and Figure 4). *P. aeruginosa* and MRSA were selected because of their high prevalence among wound infections, as well as their ability to develop resistance to available treatments [27,28]. While the current antimicrobial agents are effective against these types of infections, standard therapies may be insufficient when dealing with complex *P. aeruginosa* and MRSA infections, given the rise in antimicrobial resistance among these pathogens.

#### 3.3.1. Antimicrobial Effect on *P. aeruginosa*

Overall, *P. aeruginosa* was more susceptible to the treatment of NO-fibers than MRSA, as measured in OD at 600 nm. Such differences in sensitivity were expected, since Gram-negative bacteria are more susceptible to antimicrobial agents than Gram-positive bacteria. These differences can be attributed to differences in the cell wall thickness between the two types of bacteria [28]. Gram-negative *P. aeruginosa* has a considerably thinner cell wall in comparison to Gram-positive MRSA. Thus, the ability of NO to penetrate the cell wall and enter the bacterial cell would presumably be easier for *P. aeruginosa* than MRSA. Consequently, *P. aeruginosa* should be more susceptible to NO in comparison to MRSA, which was confirmed by antimicrobial studies.

After 24 h of NO-fiber treatment, we observed that NO-fibers inhibited the growth of *P. aeruginosa*, which was measured as optical density at 600 nm, compared to untreated *P. aeruginosa* (Figure 3). As shown in Figure 3B, the viability of *P. aeruginosa* was decreased to 67%, 15%, and 29% with PROLI-, DPTA-, and DETA-PVP NO-fibers, respectively, after 24 h. DPTA-PVP and DETA-PVP NO-fibers were more effective in preventing the growth of *P. aeruginosa*. This is possibly because of the sustained NO release by PVP, along with the long half-life of DPTA– and DETA–NONOates. The continuous release of NO is believed to be crucial in the treatment against *P. aeruginosa*, due to the presence of efflux pumps in the bacterium’s outer membrane, periplasm, and cytoplasm membrane [29]. Consequently, NO levels within the cytoplasm are significantly reduced compared to the total amount of NO released from NO-fibers.

For this reason, the sustained release of NO is essential, in order to overcome the effects of the efflux pumps and ensure that the NO concentration within the cytoplasm remains at a level capable of inducing antimicrobial effects. When treated with PROLI-EC NO-fiber, the viability of *P. aeruginosa* decreased to 46%, while DPTA- and DETA-EC NO-fibers decreased the viability to 65% and 57%, respectively. Although PVP NO-fibers showed a better antimicrobial effect, PROLI-EC NO-fiber treatment was more effective than PROLI-PVP (Figure 3D). It is likely that the rapid NO release kinetics of EC NO-fibers exert less-effective antimicrobial effects on *P. aeruginosa* than the sustained release of NO from PVP. Since the amount of NO released from EC NO-fibers remained constant after 2 h, *P. aeruginosa* bacteria were able to easily reduce the NO concentration within the cytoplasm via efflux pump mechanisms, consequently preventing the induction of antimicrobial effects. Thereby, our results indicated the importance of efflux pumps in the antibiotic resistance mechanism of *P. aeruginosa*.

#### 3.3.2. Antimicrobial Effect on MRSA

In evaluating the antimicrobial effect on MRSA, we observed that all the NO-fibers showed inhibitions of MRSA growth compared to untreated MRSA, as presented in Figure 4. In Figure 4B, it showed that PROLI-PVP NO-fibers were the least effective, as the viability was determined to be 86%, while MRSA treated with both DPTA- and DETA-PVP NO-fibers showed about 60% viability. We also did not observe differences in antimicrobial effect among PVP and EC NO-fibers on MRSA (Figure 4B,D). However, PROLI-EC NO-fibers were shown to be the most effective against MRSA, as treatment resulted in an approximately 50% decrease in MRSA viability over 24 h (Figure 4D). The antimicrobial effect of PROLI-EC NO-fibers can be attributed to the rapid release of NO, along with the short half-life of PROLI–NONOate. As discussed in Section 3.2, PROLI-EC NO-fibers exhibited rapid release among all the NO-fibers. This rapid influx of high concentrations of NO may be crucial in penetrating the thick cell wall of MRSA. More importantly, MRSA is one of the few bacterial strains that express their own nitric oxide synthase, bNOS, which largely contributes to its innate antibiotic resistance [30,31]. bNOS allows for endogenous NO production within the bacterium, which consequently protects it against exogenous NO, by counteracting increased levels of oxidative stress. In addition, induction of a flavohemoprotein (Hmp) and L-lactate dehydrogenase, upon exposure to NO, help MRSA to reduce the effects of reactive nitrosative species (ROS), which are derived from exogenous NO [32,33]. From our observations, we propose that a rapid release of NO is necessary in order to counteract the bacterium’s mitigating factors and allow NO/ROS to reach a level within the bacterium, to induce antimicrobial effects.

### 3.4. In Vitro Cytotoxicity of NO-Fibers in Cells

**Figure 5:**
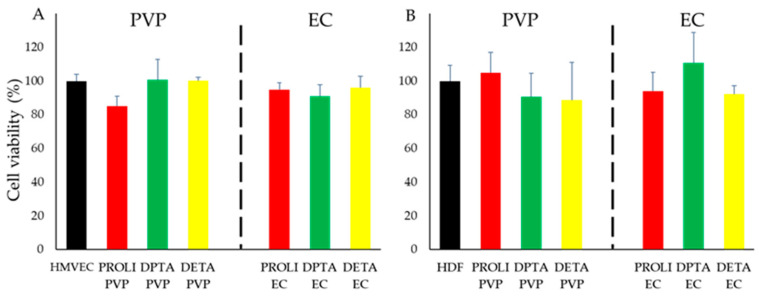
In vitro evaluation of the cytotoxic effect of NO-fibers on HMVEC and HDF cells. Cell viability was measured using alamarBlue assay after 24 h of incubation with PVP and EC NO-fibers. (**A**) Percent viability of HMVEC with respect to untreated HMVEC; (**B**) percent viability of HDF with respect to untreated HDF. Data are shown as mean ± SD.

To ensure the safety, we evaluated the cytotoxicity of NO-fibers in cell culture models, human microvascular endothelial cells (HMVECs), and human dermal fibroblast cells (HDF) (Figure 5). We treated cells with NO-fibers and determined the cell viability after 24 h by alamarBlue assay. The viability was calculated as a percentage with respect to the untreated control group.

The toxicity of NONOates is not fully established, even though NONOates have been used in human studies as a treatment for acute respiratory distress syndrome [34,35]. Another study reported the potential cytotoxicity of NONOates, caused by the accumulation of the metabolites of NONOates [9,35].

Polymeric delivery vehicles have been shown to overcome any concerns of NONOate toxicity towards human cells, as NONOate incorporating polymeric delivery vehicles showed no toxicity towards human fibroblast cells at concentrations necessary for antimicrobial activity [36]. The biocompatibility of both polymers used in this study, PVP and EC, are well characterized, thus no cytotoxic effects were expected [14,15].

We selected HMVECs and HDFs for the evaluation of toxicity, due to the prominent role of each cell type in wound healing [37,38]. Specifically, the proliferation of HMVECs and HDFs are crucial aspects of NO’s wound healing mechanism. In our study, we observed at least 80% viability in both HMVECs and HDFs following the exposure to NO-fibers (Figure 5A,B). We did not find statistical significances compared to the untreated control groups. Hence, these results showed that all the NO-fibers did not have a significant toxic effect on HMVECs and HDFs over 24 h.

### 3.5. Evaluation of Long-Term Stability of NO-Fibers

**Figure 6:**
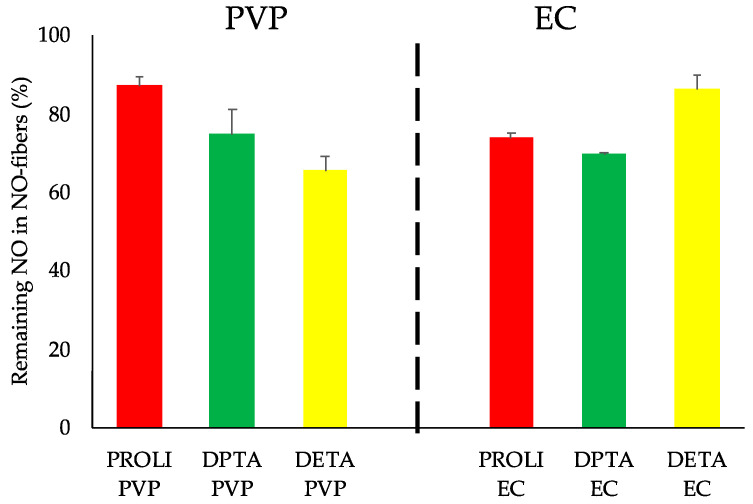
The evaluation of the long-term stability of NO-fibers after 12 months. The total NO contents from NO-fibers were compared to the total NO contents measured 12 months prior and presented in percentage. DETA-PVP NO-fibers showed the greatest loss of NO while PROLI-PVP NO-fibers and DETA-EC NO-fibers showed the highest retention of the initial NO contents over 12 months storage. *n* = 3 data is shown as mean ± SD.

We evaluated the long-term stability of NONOates in NO-fibers by analyzing the total NO content. NO-fibers in a dry state were stored in sealed containers at room temperature for 12 months before the analyses. Using the colorimetric assay, we measured the total content of NO, and the obtained values were compared to the initial total content of NO (Equation (3)). The evaluation and comparison of NO content showed that NO-fibers retained at least 65% of NO-releasing NONOates after 12 months of storage (Figure 6). Among PVP NO-fibers, PROLI NO-fibers exhibited the greatest retention, with 87% of its initial NO content remaining. DPTA NO-fibers and DETA NO-fibers retained slightly less with 75% and 65% of their initial NO content, respectively. For EC NO-fibers, DETA EC exhibited the greatest storage capacity, maintaining 86% of its initial NO content, while PROLI EC maintained 74% and DPTA EC maintained 70%. At this point, we did not find any correlation between the NO release kinetics and the long-term stability of each NO-fiber. Additionally, no discernable differences in stability were noted between biopolymers or among NONOates.

As previously mentioned, the high reactivity of NONOates with moisture is a challenge in the storage and delivery of NO. Therefore, the use of biopolymers was advantageous to stabilize NONOates, not only to prevent the premature NO release, but also to protect NONOates from moisture in the atmosphere. These results showed that the NO-fiber serves as a suitable storage platform for NONOates, even at room temperature.

## 4. Conclusions

In this study, we demonstrated the feasibility of the electrospinning process to fabricate NO-releasing polymeric nanofibers. NO-fibers were fabricated from biopolymer solutions of PVP and EC, and the following three different NONOate derivatives: PROLI–, DPTA–, and DETA–NONOate. We showed that electrospun NO-fibers were effective NO delivery platforms, as they prevented the premature release of NO, and released NO over 24 h in vitro. In addition, the release kinetics of NO-fibers were found to be polymer- and NONOate-dependent. We also studied the long-term stability of NONOates in NO-fibers over 12 months. All the NO-fibers retained at least 60% of their original NO content, thus demonstrating the capability of NO-fibers to effectively store NO. Moreover, NO-fibers inhibited the growth of *P. aeruginosa* and MRSA in vitro, and showed minimal toxicity to HDFs and HMVECs. Although further studies to evaluate in vitro antibiofilm effects of NO-fibers and the antimicrobial effect on skin infection animal models are warranted, we anticipate that NO-fibers will be an effective therapy for treating skin infections involving antibiotic-resistant organisms. NO-fiber application requires minimal effort and allows for infrequent dosing, both of which would be extremely useful for physicians when managing acute skin infections.

## Figures and Tables

**Table 1:** Total NO content and encapsulation efficiency for PVP and EC NO-fibers prepared with various NONOates in this study.

Biopolymers	PVP		EC	
NONOates	Total NO Content (μmol/mL)	Encapsulation Efficiency (%)	Total NO Content (μmol/mL)	Encapsulation Efficiency (%)
PROLI NO-fibers	1.6 ± 0.07	17	2.8 ± 0.04	30
DPTA NO-fibers	4.6 ± 0.3	37	6.7 ± 0.2	54
DETA NO-fibers	4.8 ± 0.25	46	8.1 ± 0.9	78

## Data Availability

The data presented in this study are available on request from the corresponding authors.
